# Novel Methanotrophs of the Family *Methylococcaceae* from Different Geographical Regions and Habitats

**DOI:** 10.3390/microorganisms3030484

**Published:** 2015-08-21

**Authors:** Tajul Islam, Øivind Larsen, Vigdis Torsvik, Lise Øvreås, Hovik Panosyan, J. Colin Murrell, Nils-Kåre Birkeland, Levente Bodrossy

**Affiliations:** 1 Department of Biology, University of Bergen, Thormøhlensgate 53B, Postboks 7803, 5006 Bergen, Norway; E-Mails: Vigdis.Torsvik@bio.uib.no (V.T.); lise.ovreas@bio.uib.no (L.Ø.); nils.birkeland@bio.uib.no (N.-K.B.); 2 Uni Research Environment, Thormøhlensgate 49B, 5006 Bergen, Norway; E-Mail: Oivind.Larsen@uni.no; 3 Department of Microbiology, Plant and Microbe Biotechnology, Yerevan State University, A. Manoogian 1, 0025 Yarevan, Armenia; E-Mail: hpanosyan@yahoo.ca; 4 School of Environmental Sciences, University of East Anglia, Norwich Research Park, Norwich, NR4 7TJ, UK; E-Mail: J.C.Murrell@uea.ac.uk; 5 Commonwealth Scientific and Industrial Research Organization (CSIRO), Marine and Atmospheric Research and Wealth from Oceans National Research Flagship, Hobart, TAS 7004, Australia; E-Mail: lev.bodrossy@csiro.au

**Keywords:** *Gammaproteobacteria*, *Methylococcaceae*, methanotrophs, *Methylocaldum*, mesophilic, terrestrial

## Abstract

Terrestrial methane seeps and rice paddy fields are important ecosystems in the methane cycle. Methanotrophic bacteria in these ecosystems play a key role in reducing methane emission into the atmosphere. Here, we describe three novel methanotrophs, designated BRS-K6, GFS-K6 and AK-K6, which were recovered from three different habitats in contrasting geographic regions and ecosystems: waterlogged rice-field soil and methane seep pond sediments from Bangladesh; and warm spring sediments from Armenia. All isolates had a temperature range for growth of 8–35 °C (optimal 25–28 °C) and a pH range of 5.0–7.5 (optimal 6.4–7.0). 16S rRNA gene sequences showed that they were new gammaproteobacterial methanotrophs, which form a separate clade in the family *Methylococcaceae*. They fell into a cluster with thermotolerant and mesophilic growth tendency, comprising the genera *Methylocaldum*-*Methylococcus-Methyloparacoccus*-*Methylogaea*. So far, growth below 15 °C of methanotrophs from this cluster has not been reported. The strains possessed type I intracytoplasmic membranes. The genes *pmoA*, *mxaF*, *cbbL*, *nifH* were detected, but no *mmoX* gene was found. Each strain probably represents a novel species either belonging to the same novel genus or each may even represent separate genera. These isolates extend our knowledge of methanotrophic *Gammaproteobacteria* and their physiology and adaptation to different ecosystems.

## 1. Introduction

Aerobic methane-oxidizing bacteria (MOB) represent a unique group of prokaryotes, which play an important role in the global carbon cycle and act as an environmentally important biofilter to reduce methane gas emissions to the atmosphere [1]. These bacteria are ubiquitous in nature and have been isolated from a wide variety of environments, including wetlands, agricultural soil, forest soil, landfill soil, fresh-water, seawater, lake and marine sediments, hot springs, Arctic tundra soil, acid peat bogs, peat soil, alkaline soda lakes, and oils and tailings [2,3,4,5]. MOB can be distinguished from other microorganisms by their unique capability to use methane as sole carbon and energy source. Most extant MOB are mesophilic and neutrophilic organisms growing at a temperature range of 20–37 °C and moderate pH range of 5.0–8.0 [6]. A few thermophilic, thermotolerant and mesophilic species of gammaproteobacterial methanotrophs, with growth ranges between 20 and 67 °C, have also been cultivated and described [7,8,9,10,11,12]. The thermophilic and mesophilic verrucomicrobial methanotrophs have been isolated from acidic thermal habitats [13,14,15,16,17,18]. Proteobacterial aerobic methanotrophs have been studied extensively [19]. In the class *Gammaproteobacteria*, 14 genera of the family *Methylococcaceae* (*Methylobacter*, *Methylosphaera*, *Methylomonas*, *Methylomicrobium*, *Methylosarcina*, *Methylosoma*, *Methylovulum*, *Methylomarinum*, *Methyloglobulus*, *Methyloprofundus*, *Methylococcus*, *Methylocaldum*, *Methylogaea*, *Methyloparacoccus*) [12,20,21] and three genera of the family *Methylothermaceae* (*Methylothermus*, *Methylohalobius*, *Methylomarinovum*) are validly described [11]. In the class *Alphaproteobacteria*, five genera are described (*Methylocystis*, *Methylosinus*, *Methylocella*, *Methylocapsa* and *Methyloferula*) [22,23].

Several type I methanotrophs of the family *Methylococcaceae* isolated and detected in various permanently cold habitats [24], such as *Methylosoma difficile* [25], *Methylobacter tundripaludum* [26], *Methylomonas scandinavica* [27] and *Methylomonas paludis* [28], have been described as new species or genera. Recently, two new genera of non-thermotolerant methane-oxidizers, *Methylogaea* and *Methyloparacoccus*, were described, which clustered with the thermotolerant and moderately thermophilic clade, *Methylococcus-Methylocaldum*, based on 16S rRNA gene phylogeny [12]. Here, we report the isolation and initial characterization of three methanotrophic strains from environmental samples collected in three unique geographic regions: waterlogged rice field soil (Gazipur, Bangladesh), terrestrial methane seep pond sediments (Sylhet, Bangladesh) and warm spring sediments (Akhurik, Armenia). The strains cluster most closely in 16S rRNA gene phylogeny to the genera *Methylocaldum* and *Methyloparacoccus* of the family *Methylococcaceae*.

## 2. Materials and Methods

### 2.1. Sampling and Cultivation Conditions

Samples were collected using sterile Falcon tubes, from waterlogged rice field soil in Gazipur, Bangladesh (23°58′25″ N, 90°23′39″ E), terrestrial methane seep pond sediments in Sylhet, Bangladesh (24°58′51″ N, 92°01′43″ E), and warm spring sediments in Akhurik, Armenia (44°34′04″ N, 46°53′95″ E), and transported to Bergen, Norway, for further analysis (Table S1). For the enrichment and cultivation of mesophilic obligate methane oxidizers, a low-salt mineral medium, LMM (low-salt methanotrophic medium), was applied. LMM contained the following (g·L^−1^): KNO_3_, 0.1; MgSO_4_, 0.1; CaCl_2_·2H_2_O, 0.02; KBr, 0.01. To this medium (1 L), 100 μL of a stock solution of trace elements containing (g·L^−1^) ZnSO_4_·7H_2_O, 0.4, Na_2_EDTA, 0.25, MnCl_2_·2H_2_O, 0.02, H_3_BO_3_, 0.015, Na_2_MoO_4_·2H_2_O, 0.04, NiCl_2_·6H_2_O, 0.01, CuSO_4_·5H_2_O, 0.2, CoCl·6H_2_O, 0.05, and 100 μL of an iron stock solution (FeNaEDTA, 4.5 g·L^−1^) were added. After autoclaving, the following solutions were added to the medium: (1) 100 μL of a filter-sterilized vitamin stock solution containing (mg·L^−1^) thiamine hydrochloride, 10; nicotinic acid, 20; pyridoxamine, 10; *p*-aminobenzoic acid, 10; riboflavin, 20; biotin, 1; and cyanocobalamin (vitamin B_12_), 5; (2) 1 mL of a phosphate buffer stock solution containing (g·L^−1^) KH_2_PO_4_, 37.5, Na_2_HPO_4_·2H_2_O, 49; and (3) 100 μL of a selenite-tungstate solution containing (mg·L^−1^) NaOH, 400, Na_2_SeO_3_·5H_2_O, 6, Na_2_WO_4_·2H_2_O, 8. Finally, the pH was adjusted to 6.8 with HCl or NaOH.

For enrichment cultures, 2 g waterlogged rice field soil or 2 mL slurry from each of the terrestrial methane seep and warm spring sediments were added directly to 15 mL LMM medium in 120mL sterile serum flasks. The flasks were closed with butyl rubber stoppers and aluminum crimp seals. A mixture of methane (purity 95.5%, Yara Praxair, Oslo, Norway) and air was added aseptically through a syringe to achieve 80% methane and 20% air in the headspace. When nothing else is specified, this methane:air mixture was used in all culturing and growth experiments. The flasks were incubated at 25 °C for two weeks in the dark and without shaking. The gas mixture was replaced every five days. Growth was routinely monitored by phase-contrast microscopy (Eclipse E400 microscope, Nikon Corporation, Tokyo, Japan).

### 2.2. Isolation of Three Mesophilic Methanotrophs

The primary enrichment cultures were transferred five times in fresh LMM medium. Then, serial dilutions were made, and 100 μL of dilutions (10^−5^ to 10^−8^) were spread onto plates containing LMM medium solidified with gelrite (20 g·L^−1^; Gelzan^TM^ CM, Sigma-Aldrich Corporation, St. Louis, MO, USA). The gelrite plates were incubated for two to four weeks at room temperature (22 °C) in jars filled with methane gas and air. Individual colonies were re-streaked onto fresh plates and re-incubated under the same conditions for two to three weeks, and this process was repeated twice. One strain from each of the environments, wet rice field soil, methane seep and warm spring sediments, was isolated and designated as strains BRS-K6, GFS-K6 and AK-K6, respectively. LMM medium was used for routine cultivation at 25 °C with methane and air in the head space. The purity of cultures was assessed by phase-contrast and transmission electron microscopy (TEM, at 60 KV, Jeol JEM-1230, Tokyo, Japan), the observation of single colony growth on gelrite plates in atmosphere without methane, heterotrophic contamination tests in Luria-Bertani broth in dilution (1 to 5% v/v) and on R2A (Reasoner’s 2A agar) plates [29]. The absence of growth on these media together with microscopy observation confirmed the purity of the cultures.

### 2.3. Growth Conditions, Including Carbon and Nitrogen Sources

The ability to use various organic compounds as a carbon source were tested in liquid LMM medium supplemented with sterile substrates (acetate, pyruvate, succinate, glucose, lactate, maltose, ethanol, sorbitol and yeast extract) at a concentration of 10 mM. Growth on methanol, formaldehyde and formate was examined using LMM medium containing substrate concentrations of 0.01%, 0.05%, 0.1%, 0.25%, 0.5%, 0.75% and 1% (v/v). The cell growth of each strain was tested at temperatures of 5, 8, 10, 15, 20, 22, 25, 28, 30, 35, 37, 40 and 45 °C and pH 4.5, 5, 5.5, 6.0, 6.5, 6.8, 7.2, 7.5 and 8.0. Nitrogen sources were tested by using liquid LMM medium in which KNO_3_ (0.1 g·L^−1^) was replaced by 0.1 g·L^−1^ of NH_4_Cl, methylamine or glycine. The growth of strains was also tested in triplicate with nitrogen-free LMM medium, where N_2_ from the air (20% air in the headspace) was the only nitrogen source. Growth was examined after two weeks. The specific growth rate (μ) and the generation time of each strain growing on methane were calculated from the exponential phase of growth under optimal conditions (25 °C).

### 2.4. Naphthalene Assay, Acetylene Inhibition and Heat Resistance Tests

The naphthalene-oxidation assay for sMMO (soluble methane monooxygenase) was performed with a liquid LMM culture without copper as described by Graham *et al*. [30], and *Methylococcus capsulatus* strain Bath was applied as a positive control. The effect of acetylene was tested by adding 4% (v/v) acetylene in the headspace of three replicate flasks containing active cultures of isolates in early exponential phase growing in LMM. To verify the acetylene inhibition test, *Methylococcus capsulatus* strain Bath and *Methylacidiphilum kamchatkensis* strain Kam1 were used as positive controls under the same assay conditions [15,31]. To test for heat resistance, 5-week-old cultures of each strain were heated at 80 °C for 10 min.

### 2.5. Morphology and Electron Microscopy

Cell morphology was determined using phase-contrast and TEM microscopy. For electron microscopy, exponentially-grown cells were used to prepare ultrathin sections as described previously [15].

### 2.6. Cellular Fatty Acid Analysis

Fatty acid analysis was performed by the Identification Service of the Deutsche Sammlung von Mikroorganismen und Zellkulturen GmbH (DSMZ, Braunschweig, Germany). Active cultures of strains grown under optimal conditions were shipped to DSMZ where the sample was processed by harvesting, saponification, methylation and extraction prior to gas chromatography analysis. The fatty acid patterns were compared to the patterns stored in the fatty acid database of the Microbial Identification System (MIS) supplied by MIDI Inc. (Microbial ID, New York, NY, USA).

### 2.7. DNA Isolation, 16S rRNA Gene and Functional Genes Analyses

Genomic DNA was isolated using the GenElute Bacterial Genomic DNA kit (Sigma-Aldrich). The 16S rRNA genes were amplified as described previously [15,32] using a Veriti 96 well thermal cycler (Applied Biosystems, Carlsbad, CA, USA). The PCR products were purified and sequenced using the BigDye kit for automated DNA sequencers (ABI 3700 PE; Applied Biosystems). The genes encoding particulate methane monooxygenase (*pmoA*), soluble methane monooxygenase (*mmoX*), methanol dehydrogenase (*mxaF*) and dinitrogenase reductase H (*nifH*), as well as the ribulose-1,5-bisphosphate carboxylase/oxygenase (*cbbL*) were also PCR amplified from genomic DNA using the primers listed in Table S2. The PCR was performed using Dynazyme™ High-Fidelity DNA Polymerase (Finnzymes, Finland) and the following PCR program: initial denaturation at 95 °C for 5 min, 95 °C for 1 min, annealing at 55 °C for 1 min, extension at 72 °C for 1 min (30 cycles) and a final extension at 72 °C for 7 min. The reactions were performed in 50µL mixtures in 0.3mL microtubes. *Methylococcus capsulatus* strain Bath DNA and MilliQ water were used as the positive control and the negative control, respectively. After sequencing analysis, the 16S rRNA gene and the deduced PmoA amino acid sequences [33] were compared to available sequences in the GenBank database using the NCBI tools, BLASTn and BLASTp. 16S rRNA gene sequences and deduced amino acid sequences of PmoA were aligned using the CLUSTAL W algorithm as implemented in the MEGA6 software package [34]. To confirm phylogenetic affiliation, trees of the 16S rRNA gene sequences were constructed using the neighbour joining, maximum likelihood, minimum evolution and maximum parsimony methods, and evolutionary distances were computed using Kimura 2-parameter model, Jones–Taylor/Thornton method or the Dayhoff model, which are also implemented in MEGA6 software. The tree topologies were decided by 1000 bootstrap replications.

### 2.8. Accession Numbers

Nucleotide sequences determined in this study were deposited in the GenBank database. The accession numbers are: for strain BRS-K6: KP272133 (16S rRNA), KP870204 (*pmoA*), KP870207 (*mxaF*) and KP870210 (*nifH*); for strain GFS-K6: KP272134 (16S rRNA), KP870205 (*pmoA*) and KP870208 (*mxaF*); for strain AK-K6: KP272135 (16S rRNA), KP870206 (*pmoA*), KP870209 (*mxaF*) and KP870211 (*nifH*).

## 3. Results and Discussion

### 3.1. Isolation of Three Novel Methanotrophs of the Family Methylococcaceae

Three novel methanotrophic strains, BRS-K6, GFS-K6 and AK-K6, were isolated from three different geographical regions and habitats. After inoculation with waterlogged soil and sediment samples and incubation with methane as the only carbon source, turbidity was observed after two weeks, and microbial growth was confirmed by phase-contrast microscopy. Growth occurred at 25 °C, and large rod cells were predominant during the first enrichments. During five successive sub-culturing in fresh LMM medium, the abundance of large rod/coccus cells progressively increased. To recover these cells from sub-cultures, serial dilution were made and spread onto LMM gelrite plates. Large white colonies were visible after two weeks’ incubation with all three samples. Three colonies from one plate of each environmental enrichment were picked and re-streaked on fresh plates and re-incubated. Finally, three pure colonies from each enrichment were transferred to fresh LMM medium and grown with methane or methanol as the sole carbon and energy source. Growth of these isolates was neither observed in the absence of methane, nor methanol, nor in the presence of methane and methanol under anaerobic conditions, indicating that they were obligate aerobic methanotrophs. Vitamins were required for growth.

### 3.2. Morphological and Physiological Properties

**Figure 1:**
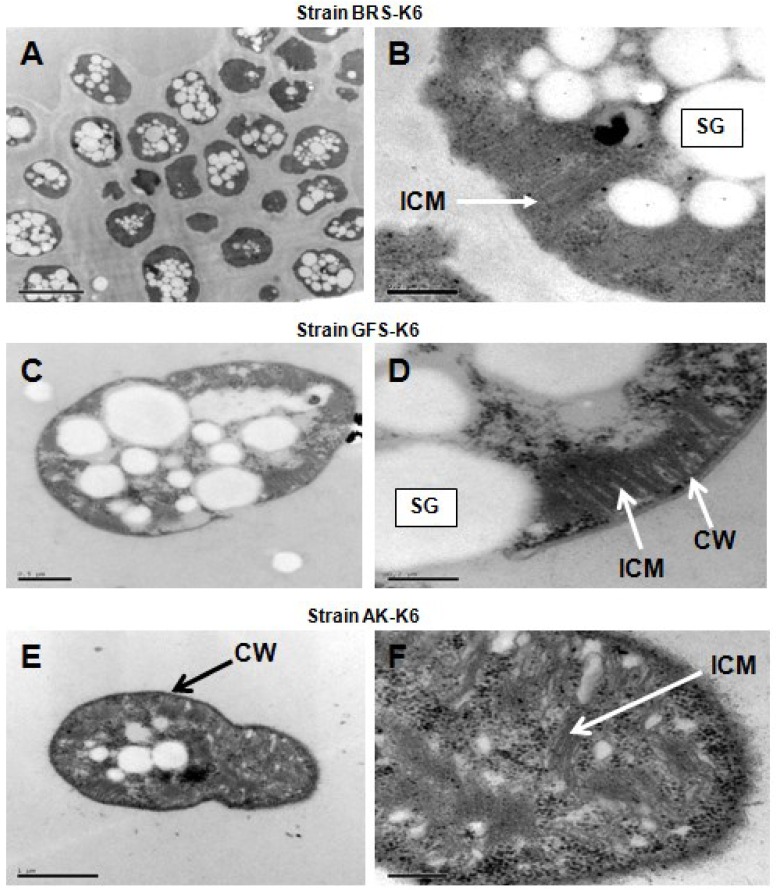
Transmission electron micrographs of thin sections of the three strains, which illustrates bundles of vesicular disks that are typical of gammaproteobacterial methanotroph intracytoplasmic membranes (ICM). Storage granules (SG) and a typical Gram-negative cell wall (CW). Bars, 2 μm (**A**), 0.2 μm (**B**, **D**, **F**), 0.5 μm (**C**), 1 μm (**E**).

The cell morphology of the isolates, BRS-K6, GFS-K6 and AK-K6, grown in liquid culture in either exponential or stationary phase were short rods with a length of 1.5–2.2 μm and a diameter of 0.5–1.5 μm (Figure 1). One week old colonies (3 mm in diameter) of all of the strains on LMM with gelrite were white and round, and no change in colour was observed after two to three weeks. They also formed white colonies on the same medium solidified with agar. Cells of all three strains were Gram-negative, divided by symmetrical binary fission and were non-motile and occurred individually or as pairs (Figure 1A,C,E), and no larger aggregations of cells (tetracocci or larger clumps) were seen. *Azotobacter*-type cysts were not detected by TEM or phase-contrast microscopy, and cells were not heat resistant. TEM analysis of ultrathin sections showed typical Gram-negative cell wall structures and the presence of the intracytoplasmic membrane (ICM) system appearing as bundles of vesicular disks typical of type I gammaproteobacterial methanotrophs (Figure 1B,D,F). Storage granules (probably poly-β-hydroxybutyrate) were also observed. Cell morphology depended on the growth temperature, as cells grown at 32–35 °C became elongated.

All three strains utilized only methane or methanol (0.05%–0.50% *v*/*v*) as the sole carbon and energy source. Cell growth was not supported by any of the multicarbon substrates tested. All strains used nitrate, ammonia or atmospheric N_2_ as a nitrogen source. Better growth was observed with LMM containing KNO_3_ than NH_4_Cl. The temperature growth range was 8–35 °C (optimum 25–28 °C), and the pH range was 5.0–7.5 (optimum 6.4–7.0). The specific growth rates of strains BRS-K6, GFS-K6 and AK-K6 were estimated to be 0.057, 0.049 and 0.053 h^−1^, respectively. Strains did not require excess NaCl for growth in LMM medium, but showed better growth in medium supplemented with 0.1% NaCl (w/v). No growth was observed at concentrations above 0.5% NaCl (w/v). Therefore, strains were neutrophilic and mesophilic obligate methane oxidizers. Major characteristics of these strains compared to other related methanotrophic genera are given in Table S3. The addition of acetylene in the headspace blocked further growth, indicating the presence of methane monooxygenase typical for MOB [15,35]. The naphthalene-oxidation assay [2] for sMMO was negative for all three isolates.

### 3.3. Phospholipids Fatty Acids

Strains BRS-K6, GFS-K6 and AK-K6 had phospholipid fatty acids profiles different from those of known mesophilic type I methanotrophs (Table S4). The predominant fatty acids were C16:1ω7c (57.93%–69.41%) and C16:1ω5c (11.39%–30.02%). The amounts of C16:0 (4.72%–11.37%) and C14:0 (3.73%–8.43%) were comparable to other genera of mesophilic methanotrophs [9,12]. A high level of C16:1ω7c is also found in *Methyloparacoccus* and *Methylogaea*, but not in *Methylocaldum* [36]. The fatty acid C16:1ω5c was not reported for *Methylogaea* and *Methylocaldum*, whereas small amounts were found in *Methyloparacoccus* (4.2%), *Methylobacter* (7% ± 1%) and *Methylomonas* (4% ± 2%) [12]. Relatively low amounts of the unsaturated fatty acids C14:0, C15:0 and C16:0 3OH were also detected. 

The fatty acid profiles of strains BRS-K6, GFS-K6 and AK-K6 are evidently distinctive from other characterized gammaproteobacterial MOB.

### 3.4. 16S rRNA, Functional Genes and Phylogenetic Analyses

**Figure 2:**
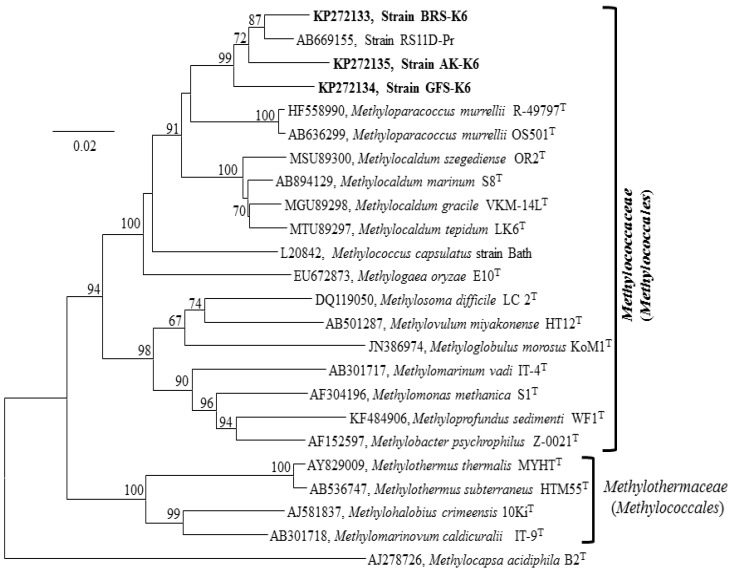
16S rRNA gene-based neighbour joining tree showing the phylogeny of three isolates, BRS-K6, GFS-K6 and AK-K6 (indicated in bold), relative to other methanotrophic strains of the class *Gammaproteobacteria* using the MEGA6 software package. Bootstrap values (percentages of 1000 data resamplings) ≥60% are shown at each node. The type II methanotroph *Methylocapsa acidophila* (AJ278726), of the class *Alphaproteobacteria*, was used as an outgroup. GenBank accession numbers are given in front of the name of respective isolates. The scale bar represents 0.02 substitutions per nucleotide position.

Nearly complete 16S rRNA gene sequence of strains BRS-K6 (1433 bp), GFS-K6 (1438 bp) and AK-K6 (1412 bp) were obtained. Neighbour joining (Figure 2), minimum evolution (Figure S1) and maximum likelihood (Figure S2) trees of 16S rRNA gene sequences gave a congruent topology. The phylogenetic analysis clearly showed that these strains comprised a new clade within the *Methylococcus-Methylocaldum-Methylogaea***-***Methyloparacoccus* cluster (Figure 2). Pairwise nucleotide sequences similarity values showed that all three strains shared between 90% and 93% 16S rRNA gene sequence identity with the genera *Methyloparacoccus* (92.9%–93.8%), *Methylocaldum* (92.2%–93.1%), *Methylococcus* (91.0%–92.0%) and *Methylogaea* (90.8%–91.4%) (Table 1). The strains shared the highest 16S rRNA gene identity (95.4%–97.8%) with the rice paddy field isolate, RS11D-Pr [37].

**Table 1:** Pairwise sequence alignment analysis of 16S rRNA sequences shows identity between BRS-K6, GFS-K6, AK-K6 and other related methane-oxidizing bacteria (MOB) [38]. Values are given as a percentage.

Strains	BRS-K6	GFS-K6	AK-K6
BRS-K6	100	-	-
GFS-K6	95.1	100	-
AK-K6	95.5	94.8	100
RS11D-Pr ^a^	97.8	95.4	96.0
*Methyloparacoccus murrellii* R-49797^T^	93.8	93.0	92.9
*Methyloparacoccus murrellii* OS501^T^	93.8	92.7	92.9
*Methylocaldum szegediense* OR2^T^	93.1	92.8	93.2
*Methylocaldum marinum* S8^T^	92.8	93.9	91.1
*Methylocaldum tepidum* LK6^T^	92.6	92.8	92.2
*Methylocaldum gracile* VKM 14L^T^	92.5	92.8	92.9
*Methylococcus capsulatus* strain Bath	92.2	91.2	91.0
*Methylogaea oryzae* E10^T^	91.4	91.4	90.8

^a^ A gammaproteobacterial methanotroph of the family *Methylococcaceae* isolated and reported from rhizosphere soil [37].

**Figure 3:**
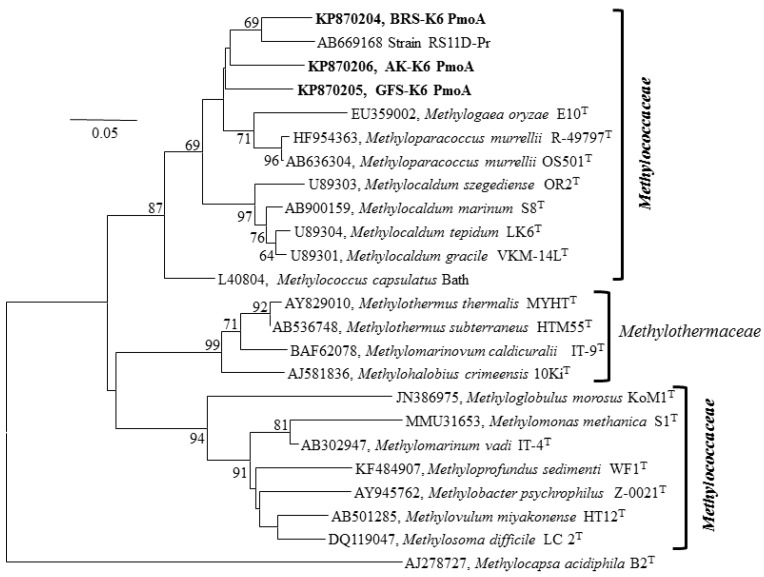
Neighbour joining tree (1000-replicate bootstrapping) based on the deduced PmoA amino acid sequences of strains BRS-K6, GFS-K6 and AK-K6 and from other cultured methanotrophs of the families *Methylococcaceae* and *Methylothermaceae*. The type II methanotroph *Methylocapsa acidophila* (AJ278727), of the class *Alphaproteobacteria*, was used as an outgroup. GenBank accession numbers are given in front of the name of respective isolates. Bar, 0.05 substitutions per amino acid position.

To confirm the presence of *pmoA* (encoding the large subunit of particulate methane monooxygenase and a key indicator gene for MOB; [39]), *mxaF* (encoding the α subunit of the methanol dehydrogenase and a marker gene of both methanotrophs and methylotrophs; [40]), *nifH* (dinitrogenase reductase H; [41]) and *cbbL* (encoding the large subunit of RuBisCO; [42]) were amplified by PCR and sequenced. PCR amplification of the *mmoX* gene, encoding the soluble form of methane monooxygenase (sMMO), was negative for all three strains (Table S2). Neighbour joining (Figure 3) and minimum evolution (Figure S3) and maximum likelihood (Figure S4) trees of partial-derived PmoA amino acid sequences showed a congruent topology. The PmoA-based phylogeny suggested that the strains were all well separated from the genera *Methyloparacoccus*, *Methylocaldum*, *Methylococcus* and *Methylogaea.*

Pairwise amino acid PmoA sequences comparison values were calculated (Table S5) and showed that PmoA of strain BRS-K6 shared 95.4% similarity with PmoA from strain GFS-K6; PmoA of strain BRS-K6 shared 95.7% with PmoA from strain AK-K6; and PmoA of GFS-K6 had 96.0% similarity with PmoA from strain AK-K6. Furthermore, protein sequences analysis revealed that the PmoA from these new strains shared 95.0%–96.9%, 93.8%–95.7%, 92.6%–94.0% and 91.9.9%–93.7% similarity with PmoA from the genera *Methyloparacoccus*, *Methylocaldum*, *Methylococcus* and *Methylogaea* and 95.4%–98.1% with PmoA from the isolate RS11D-Pr. The deduced amino acid sequences of MxaF proteins from strains BRS-K6, GFS-K6 and AK-K6 showed that they shared 94.3%–95.2% similarity with MxaF from *Methylocaldum szegediense* strain O-12 (GenBank Accession No. DQ002935), 95.5%–97.6% with MxaF from *M. capsulatus* (GenBank Accession No. U70511) and 97.0%–98.8% with MxaF from *Methyloparacoccus murrellii* R-49797^T^ (GenBank Accession No. HF954364) and *Methyloparacoccus murrellii* OS501^T^ (GenBank Accession No. HF954365) (Table S6). Furthermore, analyses of the amino acid sequences of NifH protein sequences and *nifH* nucleotide sequences of strains BRS-K6 and AK-K6 showed 97.2% similarity to NifH of *M. szegediense* (GenBank Accession No. WP_026610013) using Blastp search. The *nifH* gene was also detected in strain GFS-K6 using PCR amplification (Figure S5), but the sequences were not readable.

### 3.5. Environmental Perspective

Proteobacterial methanotrophs are ubiquitous in nature and have been isolated from a wide variety of environments [3,19,43], whereas verrucomicrobial methanotrophs (mesophilic and thermophilic) have only recently been described and found only in geothermal habitats with low pH values [16,17,18]. Osaka and colleagues [44] found 16S rRNA gene clones from activated sludge (using DNA stable isotope probing), which were affiliated with the novel uncultured methanotrophs related to the genus *Methylocaldum*. The new strain BRS-K6 showed relatively high 16S rRNA gene sequence identity (>97%) with 24% of all clones from the activated sludge sample [44]. These *Methylocaldum*-like species, therefore, might be widespread in mesophilic environments, including wet rice field soil. Closely-related *pmoA* sequences have been detected in waterlogged environments, e.g., drained rice field soil [45], from rice field soils in China and the Philippines [46], from the rice microcosm [47] and activated sludge [44]. Furthermore, *Methylococcus*-related *pmoA* sequences from lake sediment (temperature 5 °C) were also detected by the m-RNA SIP metatranscriptome analysis [48], indicating that mesophilic *Methylococcus*-like methanotrophs are ecologically more diverse than previously anticipated. In the family *Methylococcaceae*, moderately thermophilic/thermotolerant *Methylococcus* spp. and *Methylocaldum* spp. [7] and mesophilic *Methylogaea* sp. [9] and *Methyloparacoccus* spp. [12] were not reported to grow below 20 °C, whereas strains BRS-K6, GFS-K6 and AK-K6 were able to grow below 15 °C, suggesting that the new isolates are capable of growing at lower temperatures, albeit with slower growth rates. The growth optimum temperature range of strains BRS-K6, GFS-K6 and AK-K6 is 25–28 °C, and this indicates that these strains are probably mesophilic methanotrophs and well adapted to the *in situ* temperatures of the sampling sites.

### 3.6. Taxonomy

Phylogenetic analysis of the 16S rRNA gene of the three strains revealed that they belong to the class Gammaproteobacteria and represent a new clade within the family *Methylococcaceae*. Hitherto, this new clade has been represented by only one isolate, RS11D-Pr, recovered from a rice paddy field [37]. According to 16S rRNA analysis, RS11D-Pr was the closest relative to our strains (Table 1, 95.4%–97.8% sequence identity). The 16S rRNA gene phylogeny (Figure 2) showed that strain BRS-K6 and RS11D-Pr most probably belong to the same species separate from the strains GFS-K6 and AK-K6. Higher relatedness of strains BRS-K6 and RS11D-Pr was also supported by the PmoA phylogeny (Figure 3). The genera *Methylocaldum* and *Methylococcus* comprise a group of thermotolerant and moderately thermophilic methane oxidizers [7,36,49]. Within the genus *Methylocaldum*, cells are pleomorphic [49]. This phenomenon was not observed in the strains BRS-K6, GFS-K6 and AK-K6. Recently, a thermotolerant marine methane oxidizer, *Methylocaldum marinum*, was described [36] that possesses soluble methane monooxygenase (*mmoX*), which was not detected in our strains. The new clade of BRS-K6, GFS-K6 and AK-K6 is clearly separate from the thermophilic-thermotolerant cluster of *Methylocaldum-Methylococcus* and shares less than 94% 16S rRNA gene sequence identity with this cluster. PmoA-based phylogenetic analysis (Figure 3, Figures S3 and S4) of the three new strains was congruent with the 16S rRNA-based phylogeny (Figure 2, Figures S1 and S2) and confirms that they represent a new group of methanotrophic Gammaproteobacteria within the family *Methylococcaceae*. The differences in 16S rRNA gene sequences between strains BRS-K6, GFS-K6 and AK-K6 and the most closely-related genera *Methyloparacoccus*, *Methylocaldum*, *Methylococcus* and *Methylogaea* are 6%–7%, 6%–9%, 8%–9% and 9%, respectively. The sequence differences were >6%, which is too large to classify our strains within the above genera.

## 4. Conclusions

The addition of vitamins facilitated the isolation of new strains of obligate methanotrophs in the family *Methylococcaceae*. We were able to exclude heterotrophic satellites through re-streaking on LMM gelrite plates. The strains were Gram-negative, strictly aerobic, rod-shaped and did not grow on multicarbon compounds or sugars. The phenotypic and genotypic characteristics, cellular fatty acid analysis and phylogenetic analysis of these strains suggest that they most probably represent a new genus. Alternatively, each novel strain may represent a species within three novel genera in the family *Methylococcaceae*. So far, mesophilic or thermotolerant methanotrophs growing below 20 °C have not been reported for members of the cluster *Methylocaldum*-*Methylococcus-Methyloparacoccus*-*Methylogaea*. To the best of our knowledge, strains BRS-K6, GFS-K6 and AK-K6 are the first described mesophilic methanotrophs within this cluster that could grow below 15 °C. The isolation and characterization of these new isolates extend our knowledge of methanotrophic *Gammaproteobacteria* associated with terrestrial methane-rich habitats. Further genomic studies of these strains are needed to provide more insight into the ecology of the methanotrophy and phenotypic distinguishing features, as well as their influential role in the global carbon cycle.

## Supplementary Files

Supplementary File 1
