# Supplementary material for: Novel Methanotrophs of the Family Methylococcaceae from Different Geographical Regions and Habitats
Source: Microorganisms. 2015 Aug 21;3(3):484–99. doi: 10.3390/microorganisms3030484 (PMC5023254; doi:10.3390/microorganisms3030484)
Supplement: Supplementary File 1 [file microorganisms-03-00484-s001.docx]

**Supplementary Information**

**Table S1.** Isolates, sampling, location, pH and temperatures.

| **Isolates** | **Site/Location** | **Country** | **pH** | **Site Temperature(°C)** | **Sampling Time Month (year)** |
| --- | --- | --- | --- | --- | --- |
| BRS-K6 | Waterlogged rice field soil | Gazipur, | 6.8 | 23 | September (2006) and July (2010) |
|  | 23°58′25″ N 90°23′39″ E | Bangladesh |  |  |  |
| GFS-K6 | Horipur Terrestrial methane seep pond sediments | Sylhet, Bangladesh | 6.8 | 30 | June (2007) and July (2010) |
|  | 24°58′51″ N 92°01′43″ E |  |  |  |  |
| AK-K6 | Akhurik warm spring sediments |  |  |  | January (2009) |
|  | 44°34′04″ N 46°53′95″ E | Akhurik, Armenia | 7.0 | 30**–**37 |  |

**Table S2.** Primers for PCR amplification of functional genes of the strains BRS-K6, GFS-K6 and AK-K6.

| **Genes** | **Primer sequences (5′→3′)** | **Product (bp)** | **Results** | **Annealing Temperature (°C)** | **Reference** |
| --- | --- | --- | --- | --- | --- |
| *pmoA* | A189f: GGNGACTGGGACTTCTGG | 510 | + | 55 | [1] |
|  | Mb661: CCGGMGCAACGTCYTACC |  |  |  |  |
| *mxaF* | f1003: GCGGCACCAACTGGGGCTGGT | 558 | + | 58 | [2] |
|  | f1561: GGGCAGCATGAAGGGCTCCC |  |  |  |  |
| *nifH* | NifHf: GGHAARGGHGGHATHGGNAARTC | 389 | + | 55 | [3] |
|  | NifHr: GGCATNGCRAANCCVCCRCANAC |  |  |  |  |
| *cbbL* | McBCBBL 195F: CTGCTGACCGACCTCGACTA | 500 | + ^a^ | 58 | [4] |
|  | McBCBBL 706R: GTCACGTTGAGGTAGTGGCC |  |  |  |  |
| *mmoX* | mmoXf882: GGCTCCAAGTTCAAGGTCGAGC | 535 | − | 55 | [5] |
|  | mmoXr1403: TGGCACTCGTAGCGCTCCGGCTCG |  |  |  |  |
|  | f92: GGCTGCAGAGCTTYAMCTGGA |  |  |  |  |
|  | r1430: CGCCTCCCTCRTACTGYTCGAG | 1335 | − | 55 | [6] |

^a^ PCR amplification of the gene *cbbL* was detected in the strains BRS-K6, GFS-K6 and AK-K6, but sequencing analysis was not carried out.

**Table S3.** Major characteristics of strains BRS-K6, GFS-K6 and AK-K6 (this study) compared to phylogenetically-related methanotrophs of the class *Gammaproteobacteria*. Strains: ^1^ this study; ^2^ *Methylocaldum* spp. [7,8]; ^3^ *Methylocaldum marinum* S8^T^ [9]; ^4^ *Methylogaea oryzae* E10^T^ [10]; ^5^ *Methyloparacoccus murrellii* R-49797^T^ [11]; ^6^ *Methyloglobulus morosus* KoM1^T^ [12]. +, positive results; −, negative results; nr, not reported; nd, not determined.

| **Characteristic** | **^1^ Strains: BRS-K6/GFS-K6/AK-K6** | **^2^ *Methylocaldum* spp.** | **^3^ *Methylocaldum marinum*** | **^4^ *Methylogaea oryzae*** | **^5^ *Methyloparacoccus murrellii*** | **^6^ *Methyloglobulus morosus*** |
| --- | --- | --- | --- | --- | --- | --- |
| **Cell size (µm)** | 0.5–1.5 × 1.5–2.2 | 0.4–1.26 × 1.0–2.0 | 0.8–1.7 × 1.0–2.8 | 0.5–0.76 × 2.0–2.2 | 0.8–1.5 | 0.6 × 0.8–1.5 |
| Cell morphology | Rods | Rod-pleomorphic | Rods/coccus | Curved rods | Coccus | Short rods |
| ICM type | Type I | Type I | Type I | Type I | Type I | Type I |
| pMMO | + | + | + | + | + | + |
| sMMO | − | − | + | − | − | − |
| *mxaF* | + | + | + | nr | + | + |
| *nifH* | + | + ^a^ | + | + | − | + |
| *cbbL* | + | + | + | nr | nr | nr |
| Motility | − | + | − | + | − | − |
| **Temperature range (°C)** | **8–35** | **20–62** | **20–47** | **20–37** | **20–37** | **4–30** |
| Optimum temperature (°C) | 25–28 | 42–55 | 36 | 30–35 | 25–33 | 20 |
| pH range | 5–7.5 | 6–8.2 | 6–8 | 5–8 | 5.8–9.0 | 5.0–8.5 |
| pH optimum | 6.4–7.0 | 7.1–7.2 | 7 | 6.5–6.8 | 6.3–6.8 | 6.0–8 |
| Pigmentation | White | Brown | Brown | White | White | Red-pink |
| Cyst formation | − | + | + | − | − | − |
| Growth on N-free medium | + | nd | − | − | − | − |
| NaCl requirement | − | − | + | − | − | − |
| NaCl tolerance | 0.5% | nd ^b^ | 5% | 0.5% | 0.5% | <0.1M |
| Growth on methanol | + | − | + | + | − | + |
| DNA G + C content (mol%) ^c^ | nd | 57–59 | 59.7 | 63.1 | 65.6 | 47.7 |
| DNA G + C content (mol%) ^d^ | 59.5/57.3/60.3 | 56.7 **^e^** | 58.0 | 57.5 | 57.8 | 54 |

^a^ *nifH* gene sequences of *Methylocaldum* spp. are reported by Eshinimaev *et al.* [8]; ^b^ *Methylocaldum* strain O-12 and *Methylocaldum* strain H-11 could grow at 0.5% (w/v) NaCl [8]; ^c^ DNA G + C content was determined by HPLC [13]; ^d^ 16S rRNA, *pmoA*, *mxaF*, *nifH* and *cbbL* sequences were applied for the measurement of DNA G + C content (mol%); **^e^** 16S rRNA and *pmoA* sequences of *Methylocaldum szegediense* OR2^T^ were employed.

**Table S4.** Comparison of cellular fatty acid compositions between strains (data from this study) and other methanotrophs. Genera and species: 1, *Methylocaldum* spp. [7,8]; 2, *Methylocaldum marinum* S8^T^ [9]; 3, *Methylogaea oryzae* E10^T^ [10]; 4, *Methyloparacoccus murrellii* R-49797^T^ [11]; 5, *Methyloglobulus morosus* KoM1^T^ [12]. Values are given as the percentage of total fatty acids.

| **Fatty Acid** | **BRS-K6** | **GFS-K6** | **AK-K6** | **1** | **2** | **3** | **4** | **5** |
| --- | --- | --- | --- | --- | --- | --- | --- | --- |
| C12:0 |  |  |  | 0-0.1 |  | 2.11 |  | 0.1 |
| C14:0 | 4.61 | 8.43 | 3.73 | 2.0-2.4 |  | 5.84 | 4.7 | 0.9 |
| C15:0 |  | 0.83 | 0.52 | 2.5-3.5 |  | 1.03 | 3.2 | 0.7 |
| C15:1ω8c |  |  |  |  |  |  | 0.3 |  |
| **C16:1ω7c** ^a^ | **59.11** | **69.41** | **57.93** |  |  | **10.33** | **54.2** | **55.3** |
| **C16:1ω5c** | **30.02** | **11.38** | **26.46** |  |  |  | **4.2** | **28.7** |
| C16:1ω6c |  |  |  |  |  |  |  | 5.8 |
| C16:1ω5t |  |  |  |  |  |  |  |  |
| C16:0 | 4.72 | 8.45 | 11.37 | 63.7–65 | 59.2 | 62.05 | 23.7 | 6.8 |
| C16:1ω9c |  |  |  |  |  | 7.36 | 6.5 |  |
| C16:1 |  |  |  | 11.9–13.3 | 39.7 |  |  |  |
| C17:0*cyc* |  |  |  | 6.1–9.0 |  |  |  |  |
| iso-C16:0 3-OH |  |  |  |  |  | 3.96 |  |  |
| C16:0 3-OH | 1.54 | 1.50 |  |  |  | 2.93 | 2.6 | 1.0 |
| C20:0 |  |  |  |  |  | 2.66 |  |  |

^a^ Summed Feature 3 comprises C16:1ω7c or iso-C15:0 2-OH, which could not be separated by the MIDI System. However, C16:1ω7c is a frequent fatty acid in MOB [14].

**Table S5.** Pairwise sequence alignment analysis of PmoA protein sequences shows similarity between BRS-K6, GFS-K6, AK-K6 and other related MOB [15]. The identity of pairwise *pmoA* nucleotide sequences comparisons is shown in the parentheses. Values are given as a percentage.

| **Strains** | **BRS-K6** | **GFS-K6** | **AK-K6** |
| --- | --- | --- | --- |
| BRS-K6 | 100 | - | - |
| GFS-K6 | 95.4 (87.9) | 100 | - |
| AK-K6 | 95.7 (90.8) | 96.0 (88.8) | 100 |
| RS11D-Pr ^a^ | 98.1 (92.5) | 95.4 (87.2) | 95.7 (90.0) |
| *Methylocaldum szegediense* OR2^T^ | 93.8 (80.5) | 94.7 (80.8) | 93.8 (79.9) |
| *Methylocaldum tepidum* LK6^T^ | 93.8 (81.7) | 94.7 (82.4) | 93.9 (81.3) |
| *Methylocaldum gracile* VKM 14L^T^ | 95.7 (85.0) | 96.7 (85.2) | 95.7 (84.3) |
| *Methyloparacoccus murrellii* R-49797^T^ | 95.0 (88.3) | 95.3 (86.8) | 96.9 (90.0) |
| *Methyloparacoccus murrellii* OS501^T^ | 95.1 (88.3) | 95.4 (86.8) | 96.9 (90.3) |
| *Methylocaldum marinum* S8^T^ | 95.7 (89.0) | 96.7 (88.2) | 95.7 (90.4) |
| *Methylococcus capsulatus* strain Bath | 92.6 (84.6) | 94.0 (83.9) | 93.9 (86.1) |
| *Methylogaea oryzae* E10^T^ | 91.9 (81.9) | 93.7 (82.5) | 92.6 (80.6) |

^a^ A gammaproteobacterial methanotroph of the family *Methylococcaceae* isolated and reported from rhizosphere soil [16].

**Table S6.** Pairwise MxaF protein sequence comparisons between BRS-K6, GFS-K6,
AK-K6 and other related MOB [15]. The identity of pairwise *mxaF* nucleotide sequences comparisons is shown in the parentheses. Values are given as a percentage.

| **Strains (GenBank Accession No.)** | **BRS-K6** | **GFS-K6** | **AK-K6** |
| --- | --- | --- | --- |
| BRS-K6 (KP870207) | 100 | - | - |
| GFS-K6 (KP870208) | 97.0 (86.9) | 100 | - |
| AK-K6 (KP870209) | 98.9 (95.2) | 97.6 (88.4) | 100 |
| *Methyloparacoccus murrellii* R-49797^T^ (HF954364) | 97.0 (88.1) | 98.8 (85.5) | 97.7 (88.7) |
| *Methyloparacoccus murrellii* OS501^T^ (HF954365) | 97.0 (88.9) | 98.8 (85.7) | 97.7 (89.0) |
| *Methylocaldum szegediense* strain O-12 (DQ002935) | 95.2 (82.5) | 95.5 (83.3) | 94.3 (82.3) |
| *Methylococcus capsulatus* (U70511) | 95.5 (87.3) | 97.6 (86.3) | 96.6 (88.4) |

**
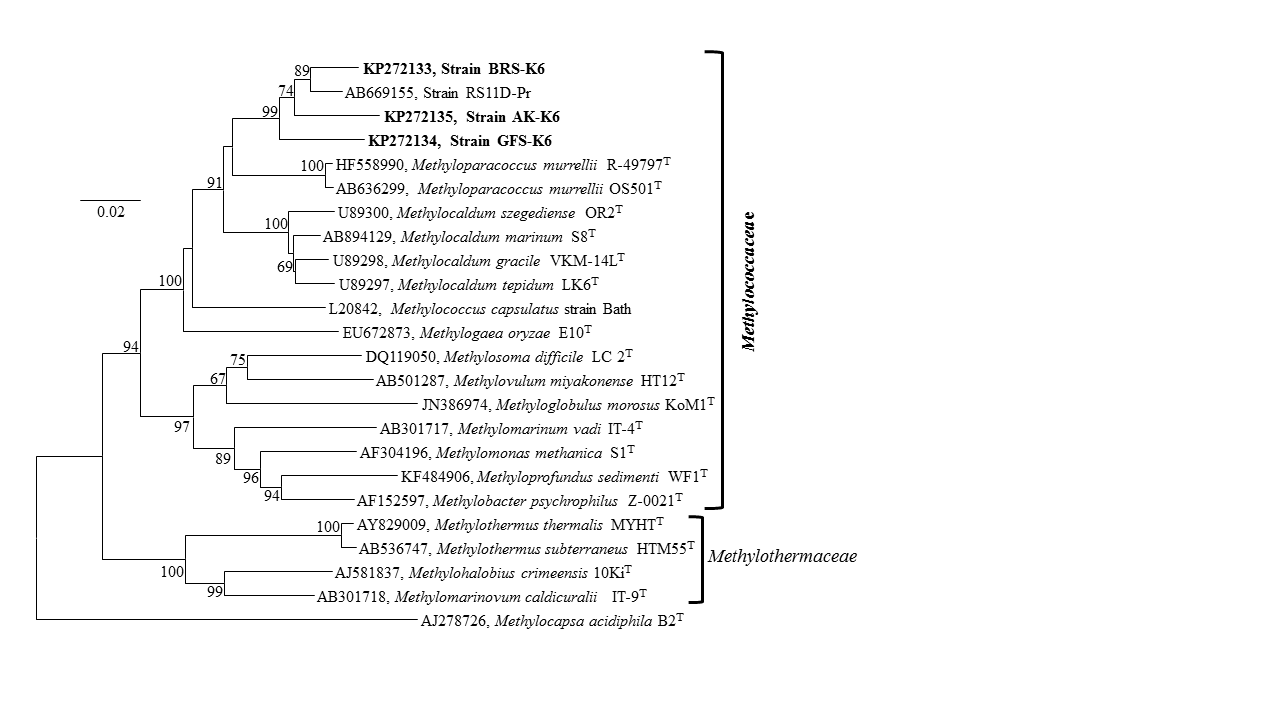
**

**Figure S1.** Minimum evolution tree, using the MEGA6 software package, of the 16S rRNA gene sequences of strains BRS-K6, GFS-K6 and AK-K6 and other described gammaproteobacterial methanotrophic isolates. The type II methanotroph *Methylocapsa acidophila* (AJ278726), of the class *Alphaproteobacteria*, was used as an outgroup. GenBank accession numbers are given in front of the names of respective isolates. Bootstrap values (1000 replicates) less than 60% are not shown. Bar, 0.02 substitutions per
nucleotide position.

**
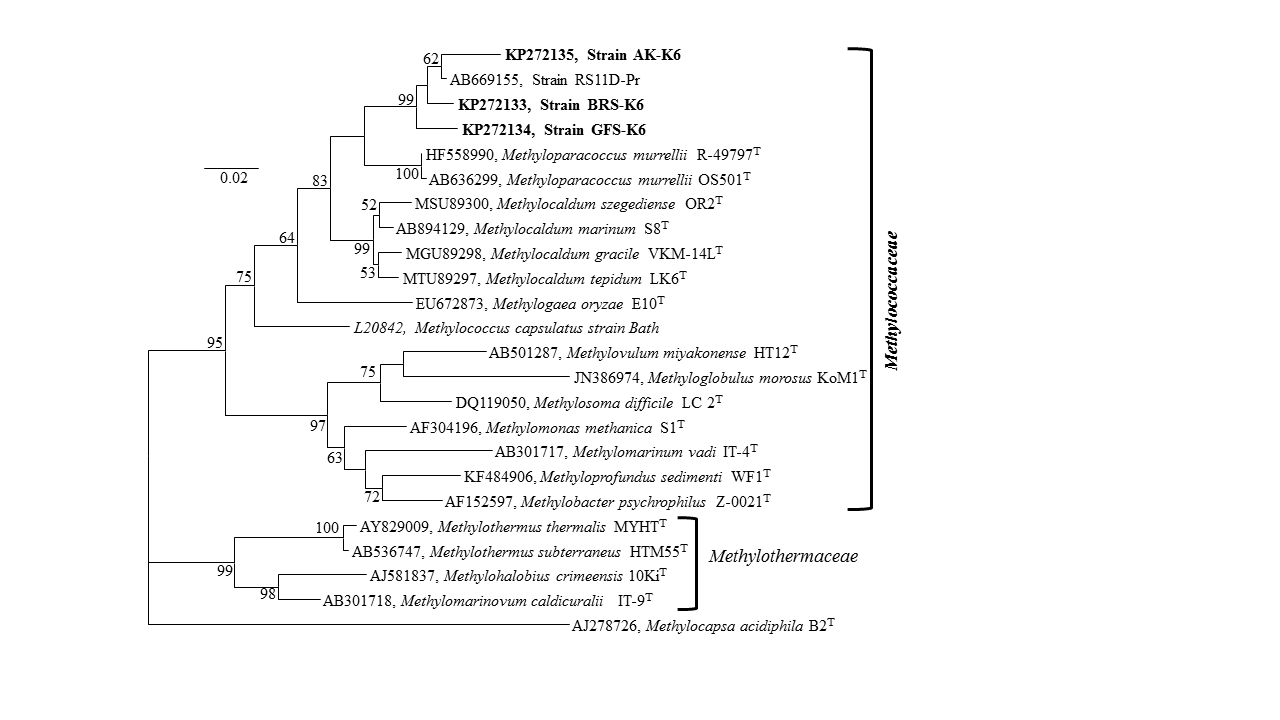
**

**Figure S2.** Maximum likelihood tree, using the MEGA6 software package, of the 16S rRNA gene sequences of strains BRS-K6, GFS-K6 and AK-K6 and other described gammaproteobacterial methanotrophic isolates. The type II methanotroph *Methylocapsa acidophila* (AJ278726), of the class *Alphaproteobacteria*, was used as an outgroup. GenBank accession numbers are given in front of the names of respective isolates. Bootstrap values (1000 replicates) less than 50% are not shown. Bar, 0.02 substitutions per
nucleotide position.

**
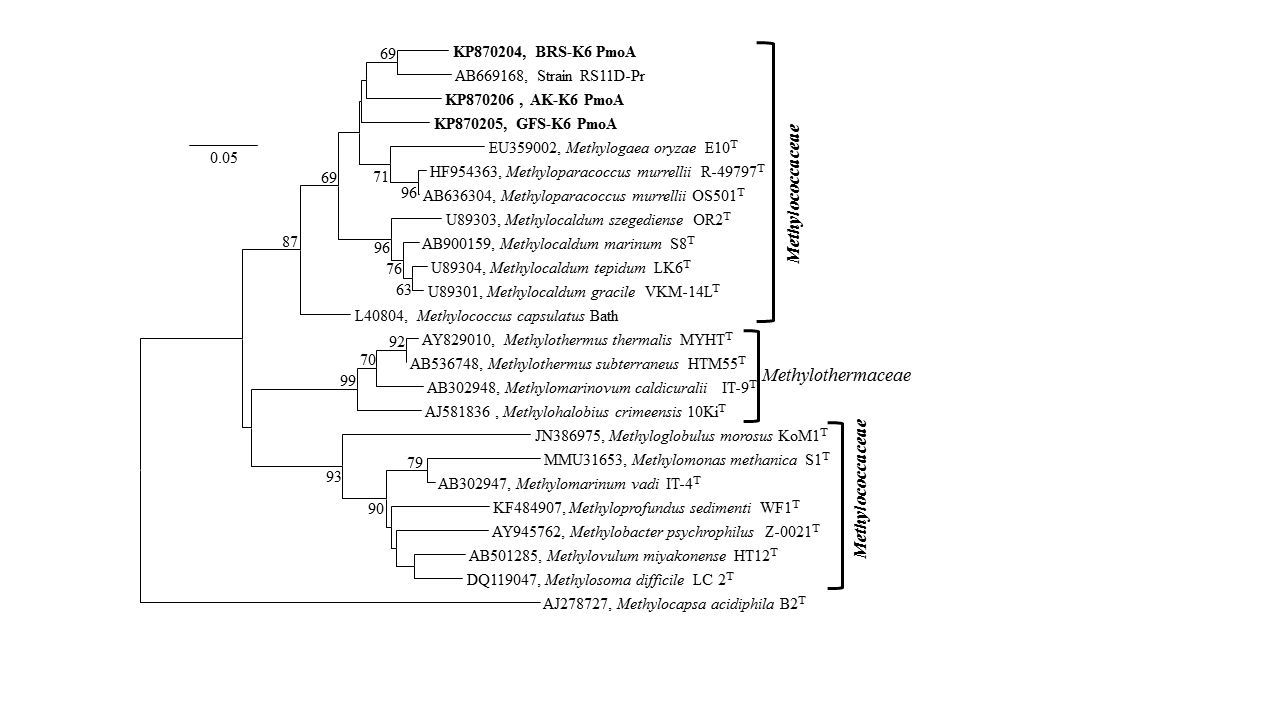
**

**Figure S3.** Minimum evolution tree, using the MEGA6 software package, based on the deduced PmoA amino acid sequences of strains BRS-K6, GFS-K6, AK-K6 and from other cultured methanotrophic Gammaproteobacteria. The type II methanotroph *Methylocapsa acidophila* (AJ278727), of the class *Alphaproteobacteria*, was used as an outgroup. GenBank accession numbers are given in front of the names of respective isolates. Bootstrap values (1000 replicates) less than 60% are not shown. Bar, 0.05 substitutions per amino
acid position.


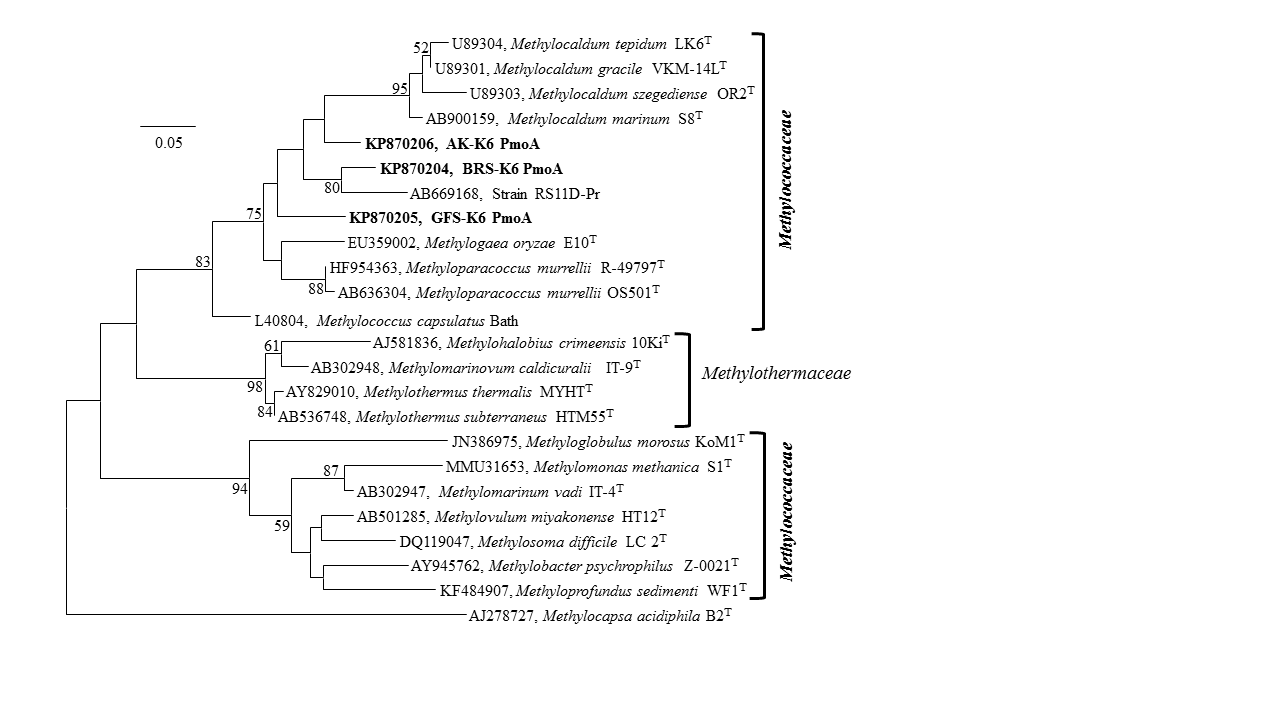


**Figure S4.** Maximum likelihood tree, using the MEGA6 software package, based on deduced PmoA amino acid sequences of strains BRS-K6, GFS-K6, AK-K6 and from other cultured gammaproteobacterial methanotrophs. The type II methanotroph *Methylocapsa acidophila* (AJ278727), of the class *Alphaproteobacteria*, was used as an outgroup. GenBank accession numbers are given in front of the names of respective isolates. Bootstrap values (1000 replicates) less than 50% are not shown. Bar, 0.05 substitutions per amino acid position.

**
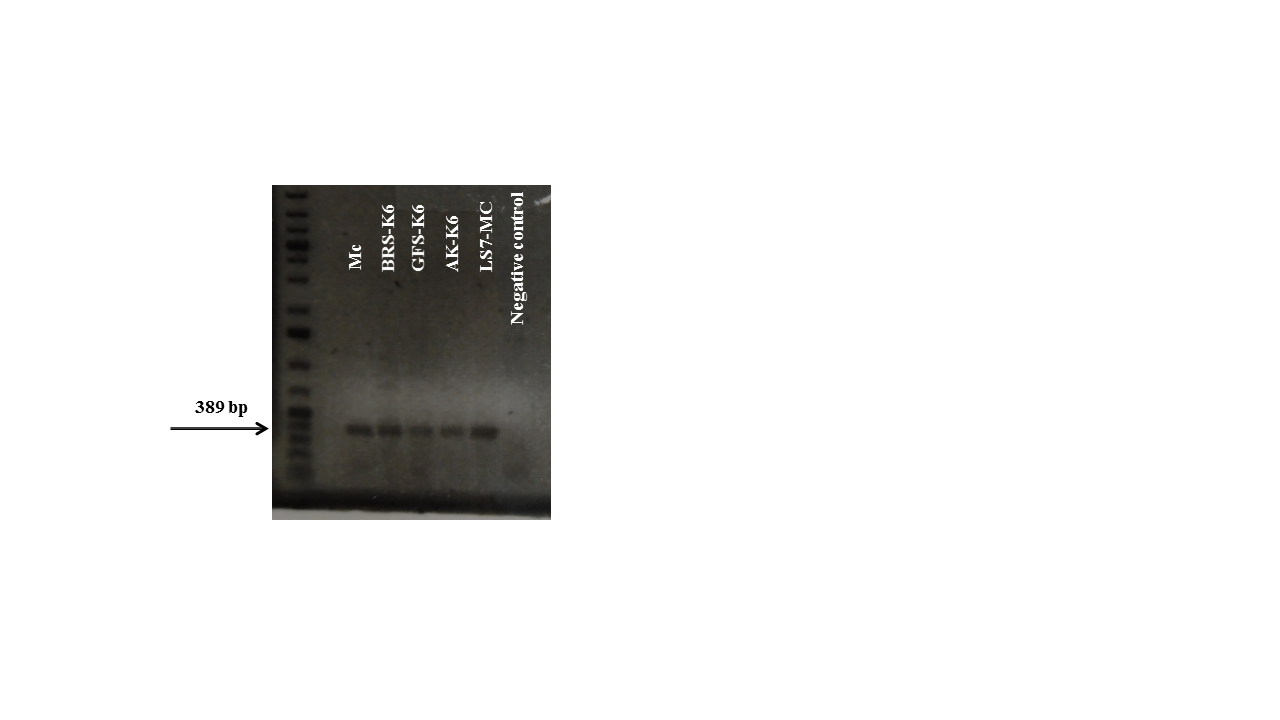
**

**Figure S5.** The PCR amplification (2% agarose gel electrophoresis) of the *nifH* gene from strains BRS-K6, GFS-K6 and AK-K6. Size are shown in base pairs (bp). The arrow indicates the presence of DNA bands as 389 base pairs. **Mc**: *Methylococcus* *capsulatus* strain Bath was applied as positive control. **LS7-MC:** A novel moderately thermophilic methanotroph which was isolated from an alkaline thermal spring in the Ethiopian Rift Valley and this isolate is not published yet.

**Reference**

1. Costello, A.M.; Lidstrom, M.E. Molecular characterization of functional and phylogenetic genes from natural populations of methanotroph*s* in lake sediments. *Appl. Environ. Microbiol.* **1999**, *65*, 5066–5074.
2. McDonald, I.R.; Murrell, J.C. The methanol dehydrogenase structural gene mxaF and its use as a functional gene probe for methanotrophs and methylotrophs. *Appl. Environ. Microbiol.* **1997**, *63*, 3218–3224.
3. Mehta, M.P.; Butterfield, D.A.; Baross, J.A. Phylogenetic diversity of nitrogenase (*nifH*) genes in
   deep-sea and hydrothermal vent environments of the Juan de Fuca Ridge. *Appl. Environ. Microbiol.* **2003**, *69*, 960–970.
4. Baxter, N.J.; Hirt, R.P.; Bodrossy, L.; Kovacs, K.L.; Embley, T.M.; Prosser, J.I.;
   Murrell, J.C. The ribulose-1,5-bisphosphate carboxylase/oxygenase gene cluster of *Methylococcus capsulatus* (Bath). *Arch. Microbiol.* **2002**, *177*, 279–289.
5. McDonald, I.R.; Bodrossy, L.; Chen, Y.; Murrell, J.C. Molecular ecology techniques for the study of aerobic methanotrophs. *Appl. Environ. Microbiol.* **2008**, *74*, 1305–1315.
6. Islam, T.; Jensen, S.; Reigstad, L.J.; Larsen, O.; Birkeland, N.K. Methane oxidation at 55 degrees C and pH 2 by a thermoacidophilic bacterium belonging to the *Verrucomicrobia* phylum. *Proc. Natl. Acad. Sci. USA* **2008**, *105*, 300–304.
7. Bodrossy, L.; Holmes, E.M.; Holmes, A.J.; Kovacs, K.L.; Murrell, J.C. Analysis of 16S rRNA and methane monooxygenase gene sequences reveals a novel group of thermotolerant and thermophilic methanotrophs, *Methylocaldum* gen. nov. *Arch. Microbiol.* **1997**, *168*, 493–503.
8. Eshinimaev, B.Ts.; Medvedkova, K.A.; Khmelenina, V.N.; Suzina, N.E.; Osipov, G.A.;
   Lysenko, A.M.; Trotsenko Yu, A. New thermophilic methanotrophs of the genus *Methylocaldum*. *Mikrobiologiia* **2004**, *73*, 530–539.
9. Takeuchi, M.; Kamagata, Y.; Oshima, K.; Hanada, S.; Tamaki, H.; Marumo, K.; Maeda, H.; Nedachi, M.; Hattori, M.; Iwasaki, W.; *et al*. *Methylocaldum marinum* sp. nov., a thermotolerant, methane-oxidizing bacterium isolated from marine sediments, and emended description of the genus Methylocaldum. *Int. J. Syst. Evol. Microbiol.* **2014**, *64*, 3240–3246.
10. Geymonat, E.; Ferrando, L.; Tarlera, S.E. *Methylogaea oryzae* gen. nov., sp. nov., a mesophilic methanotroph isolated from a rice paddy field. *Int. J. Syst. Evol. Microbiol*. **2011**, *61*, 2568–2572.
11. Hoefman, S.; van der Ha, D.; Iguchi, H.; Yurimoto, H.; Sakai, Y.; Boon, N.; Vandamme, P.;
    Heylen, K.; de Vos, P. *Methyloparacoccus murrellii* gen. nov., sp. nov., a methanotroph isolated from pond water. *Int. J. Syst. Evol. Microbiol.* **2014**, *64*, 2100–2107.
12. Deutzmann, J.S.; Hoppert, M.; Schink, B. Characterization and phylogeny of a novel methanotroph, *Methyloglobulus morosus* gen. nov., spec. nov. *Syst. Appl. Microbiol.* **2014**, *37*, 165–169.
13. Mesbah, M.; Whitman, W.B. Measurement of deoxyguanosine/thymidine ratios in complex mixtures by high-performance liquid chromatography for determination of the mole percentage guanine + cytosine of DNA. *J. Chromatogr.* **1989**, *479*, 297–306.
14. Bodelier, P.L.; Gillisen, M.J.; Hordijk, K.; Damste, J.S.; Rijpstra, W.I.; Geenevasen, J.A.;
    Dunfield, P.F. A reanalysis of phospholipid fatty acids as ecological biomarkers for *methanotrophic* bacteria. *ISME J.* **2009**, *3*, 606–617.
15. The European Molecular Biology Open Software Suite. Emboss Tools For Sequence Analysis. Available online: http://www.ebi.ac.uk/Tools/emboss/ (accessed on 15 December 2014).
16. Dianou, D.; Ueno, C.; Ogiso, T.; Kimura, M.; Asakawa, S. Diversity of cultivable methane-oxidizing bacteria in microsites of a rice paddy field: investigation by cultivation method and fluorescence in situ hybridization (FISH). *Microbes Environ. JSME* **2012**, *27*, 278–287.

© 2015 by the authors; licensee MDPI, Basel, Switzerland. This article is an open access article distributed under the terms and conditions of the Creative Commons Attribution license (http://creativecommons.org/licenses/by/4.0/).
